# Sepsis-induced immunosuppression: mechanisms, biomarkers and immunotherapy

**DOI:** 10.3389/fimmu.2025.1577105

**Published:** 2025-04-29

**Authors:** Xun Gao, Shijie Cai, Xiao Li, Guoqiu Wu

**Affiliations:** ^1^ Center of Clinical Laboratory Medicine, Zhongda Hospital, Southeast University, Nanjing, Jiangsu, China; ^2^ Department of Laboratory Medicine, Medical School of Southeast University, Nanjing, Jiangsu, China

**Keywords:** sepsis, immunosuppression, biomarkers, therapy, immunology

## Abstract

Sepsis, a life-threatening organ dysfunction resulting from a dysregulated host response to infection, initiates a complex immune response that varies over time, characterized by sustained excessive inflammation and immunosuppression. Sepsis-induced immunosuppression is now recognized as a major cause of septic death, and identifying effective strategies to counteract it poses a significant challenge. This immunosuppression results from the disruption of immune homeostasis, characterized by the abnormal death of immune effector cells, hyperproliferation of immune suppressor cells, release of anti-inflammatory cytokines, and expression of immune checkpoints. Preclinical studies targeting immunosuppression, particularly with immune checkpoint inhibitors, have shown promise in reversing immunocyte dysfunctions and establishing host resistance to pathogens. Here, our review highlights the mechanisms of sepsis-induced immunosuppression and current diagnostic biomarkers, as well as immune-enhancing strategies evaluated in septic patients and therapeutics under investigation.

## 1 Introduction

Sepsis is a life-threatening organ dysfunction resulting from a dysregulated host response to infection (1). About 48.9 million cases of sepsis were recorded globally in 2017, of which 11 million patients died of sepsis, accounting for 19.7% of global deaths (2). Although the mortality rate of sepsis has declined globally in recent years, it remains one of the most significant medical problems worldwide (3). In addition, the pathogenesis of sepsis has not been fully clarified, and clinical treatment still relies on physical therapies such as controlling infection and maintaining organ function, which results in a long treatment process and poor prognosis. Therefore, understanding the immune regulatory mechanisms of sepsis are crucial for the prevention and treatment of the disease.

**Figure 1:**
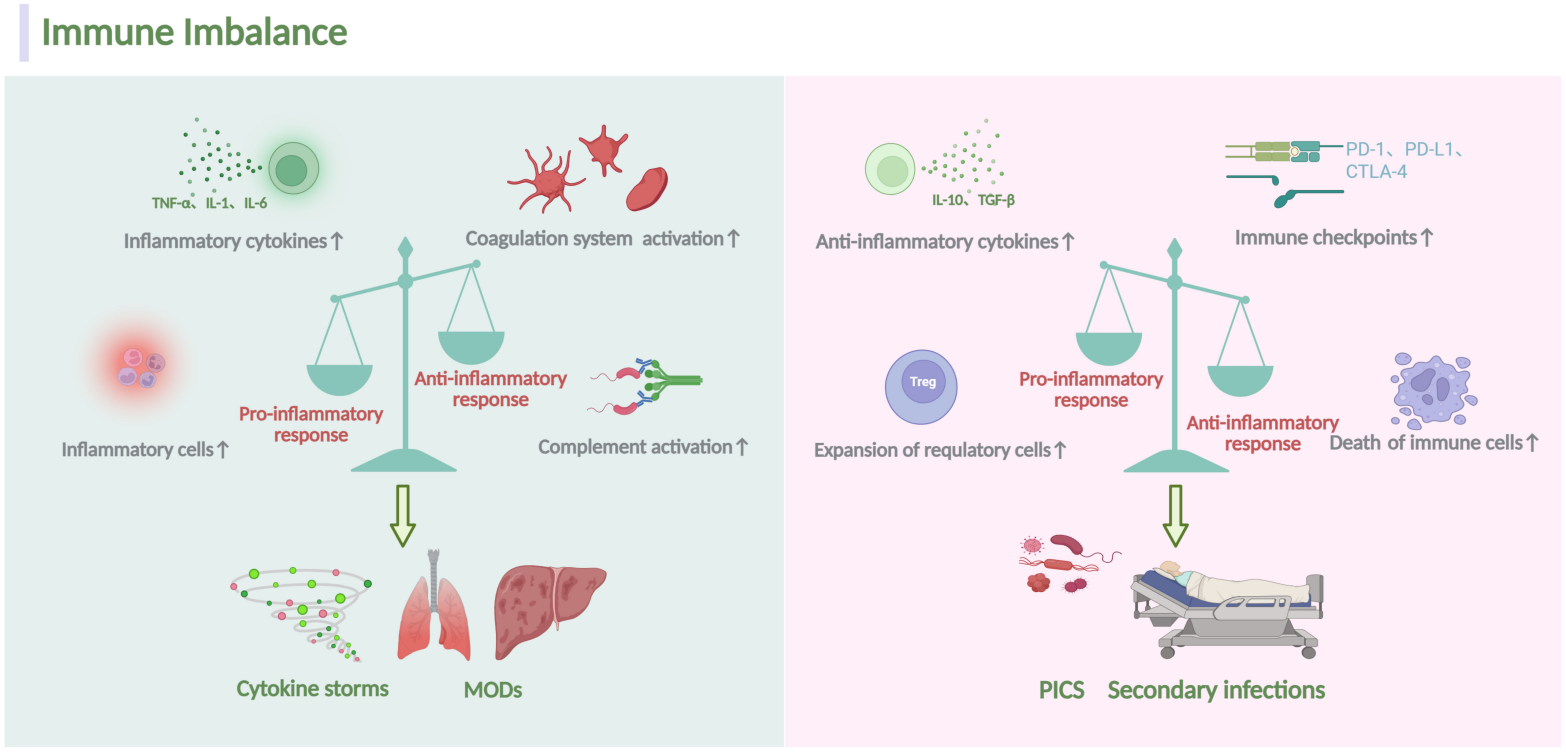
Overview of homeostatic disturbance in sepsis. During sepsis, the immune response is initiated when the host recognizes PAMPs and DAMPs involving both innate and adaptive immune system. Several mechanisms, including the proliferation of inflammatory cells, secretion of inflammatory cytokines and activation of the complement system, can escalate pro-inflammatory responses. Conversely, heightened abnormal death of immune effectors cells, production of anti-inflammatory cytokines, expansion of immunosuppressive cells and expression of specific immune checkpoints can exacerbate anti-inflammatory responses. Dominance of the pro-inflammatory response often correlates with a massive cytokine storm, leading to MODs. Conversely, the dominance of the anti-inflammatory response typically results in secondary infections and a poor prognosis. The figure was created via BioRender (https://BioRender.com).

**Figure 2:**
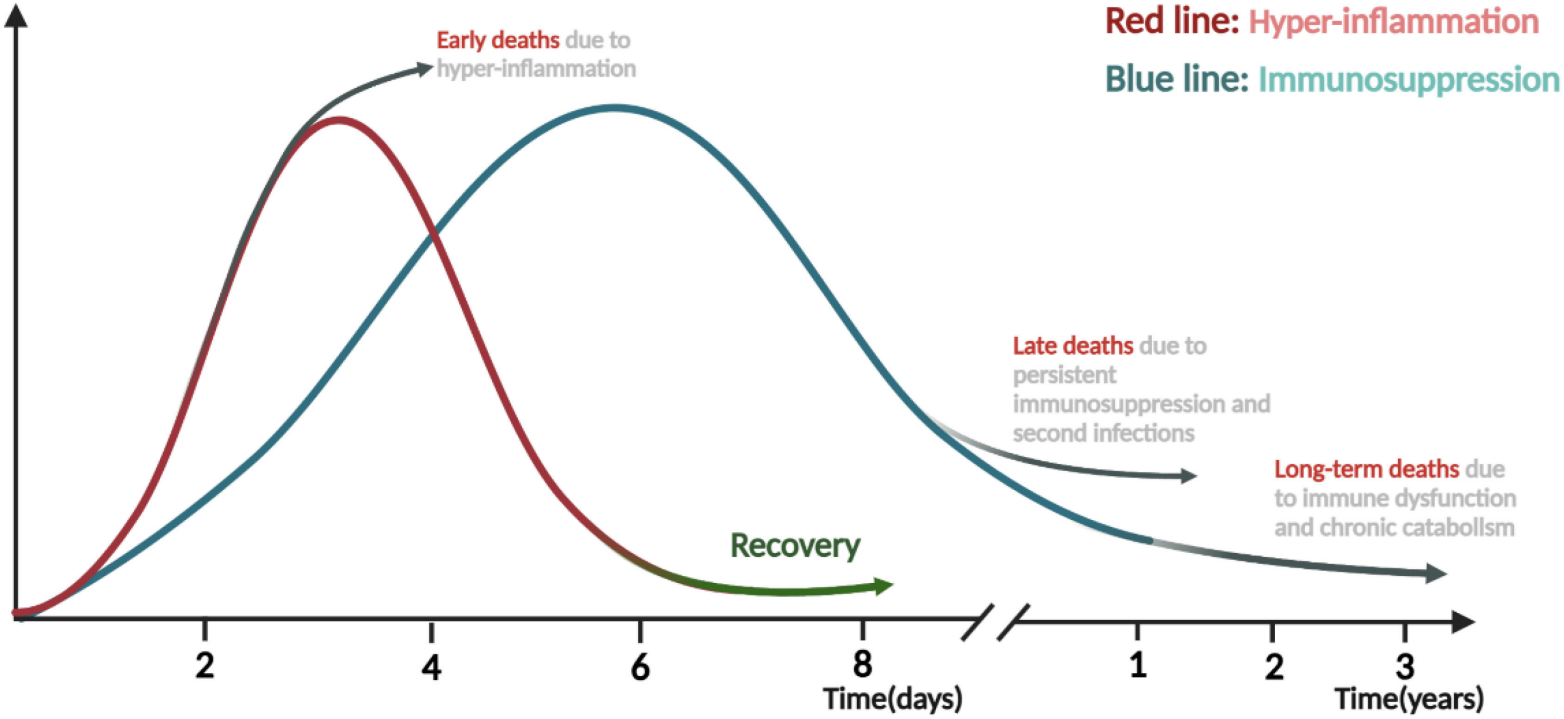
Time course and transition from hyper-inflammation to immunosuppression phase in sepsis. Both pro-inflammatory and anti-inflammatory responses are rapidly triggered after the onset of sepsis, and the transition from immune homeostasis to immune imbalance plays vital roles in the pathogenesis and progression of sepsis. The early stage is mainly dominated by the hyper-inflammation, during which the excessive “cytokine storm” often lead to early death of patients. On the contrary, if the body can restore normal immunity and rebalance, patients will enter the recovery stage or be gradually dominated by immunosuppression and suffer late or long-term death due to secondary infections, immune dysfunction and chronic catabolism. The figure was created via BioRender (https://BioRender.com).

The immune status of septic patients changes dynamically at different stages, involving complex interactions between multiple immune cells and molecular mechanisms (4). When invade, pathogens or pathogen-associated molecular patterns (PAMPs), damage-associated molecular patterns (DAMPs) may recognize pattern recognition receptors (PRRs) like RIG-I-like receptors (RLRs), Toll-like receptors (TLRs), and NOD-like receptors (NLRs), et al, on innate immune cells, triggering the innate immune responses characterized by the secretion of amounts of cytokines and chemokines, accompanied by the activation of the coagulation and complement system. An uncontrolled inflammatory response (cytokine storm) or worse still, multiple organ dysfunction syndrome (MODs) may occur as a consequence (5). If the immune system clears pathogens promptly during the early systemic inflammatory response, immune balance can be rapidly restored. However, if pathogens are not removed in time, they can lead to long-term immunosuppression, immune collapse, and even physical disabilities in patients, known as immune-suppression (6) (**Figure 1** **).** In the context of sepsis, the immune status alterations across various stages are intricate. A complex interplay between hyperinflammation and immunosuppression can manifest either sequentially or concurrently. The conventional theory posits that the early phase of sepsis is dominated by a pro-inflammatory response characterized by cytokine storm, and the early-stage mortality is attributed predominantly to MODs due to this hyper-inflammation. After the hyper-inflammation stage, the patients are either gradually recovered or transitioned into persistent immunosuppressive state characterized by immune cell exhaustion whereas this late-stage mortality is frequently associated with secondary infection. If the immunosuppression persist, patients would then suffer long-term death as a result of immune dysfunction and chronic catabolism (4, 7, 8) (**Figure 2** **).**

The excessive inflammatory responses and cytokine storms have long been considered primary contributors to the high mortality of sepsis, while therapeutic interventions targeting TNF-α or IL-6, et al, have not yielded the anticipated improvements in patient survival in the past decades (9, 10). Additionally, the use of antibiotics, fluid resuscitation, and organ support therapy have limited prognostic impact in patients with sepsis. This discrepancy between the theoretical understanding of sepsis pathophysiology and the practical outcomes of targeted therapies underscores the complexity of the disease and the need for a more nuanced approach to treatment. In recent years, the role of immunosuppression in sepsis has been increasingly emphasized. Persistent immunosuppression is characterized by excessive and aberrant immune cell death, sustained release of anti-inflammatory mediators, expansion of immunomodulatory cell populations, and upregulation of immune checkpoint molecules, which collectively compromise host defenses against pathogens (11–14). This immunosuppressive state not only increases the risk of secondary infections but also diminishes the efficacy of therapeutic interventions such as immunotherapy and antibiotics (15, 16). Clinical studies have demonstrated that persistent lymphopenia in sepsis patients is strongly associated with 28-day mortality, prolonged hospitalization, and a higher incidence of secondary infections. Notably, sepsis-associated immunosuppression is recognized as a major contributor to mortality through its role in facilitating secondary infections (4, 17). Therefore, understanding and coping with sepsis immunosuppression is crucial for improving patient prognosis and therapeutic outcomes.

Here, we summarize current advances in the mechanisms underlying sepsis-induced immunosuppression, including the dysfunction of immune effector cells, expansion of immune suppressive cells, overproduction of anti-inflammatory cytokines, and dysregulation of immune checkpoint pathways. We also comprehensively summarize the diagnostic and therapeutic biomarkers related to sepsis immunosuppression, highlighting their translational potential in guiding the development of precision therapies for septic patients. Finally, we discuss new insights in immunosuppression-targeted therapies for sepsis, offering a framework to refine clinical staging and advance evidence-based management strategies.

## 2 Mechanisms of sepsis induced-immunosuppression

**Figure 3:**
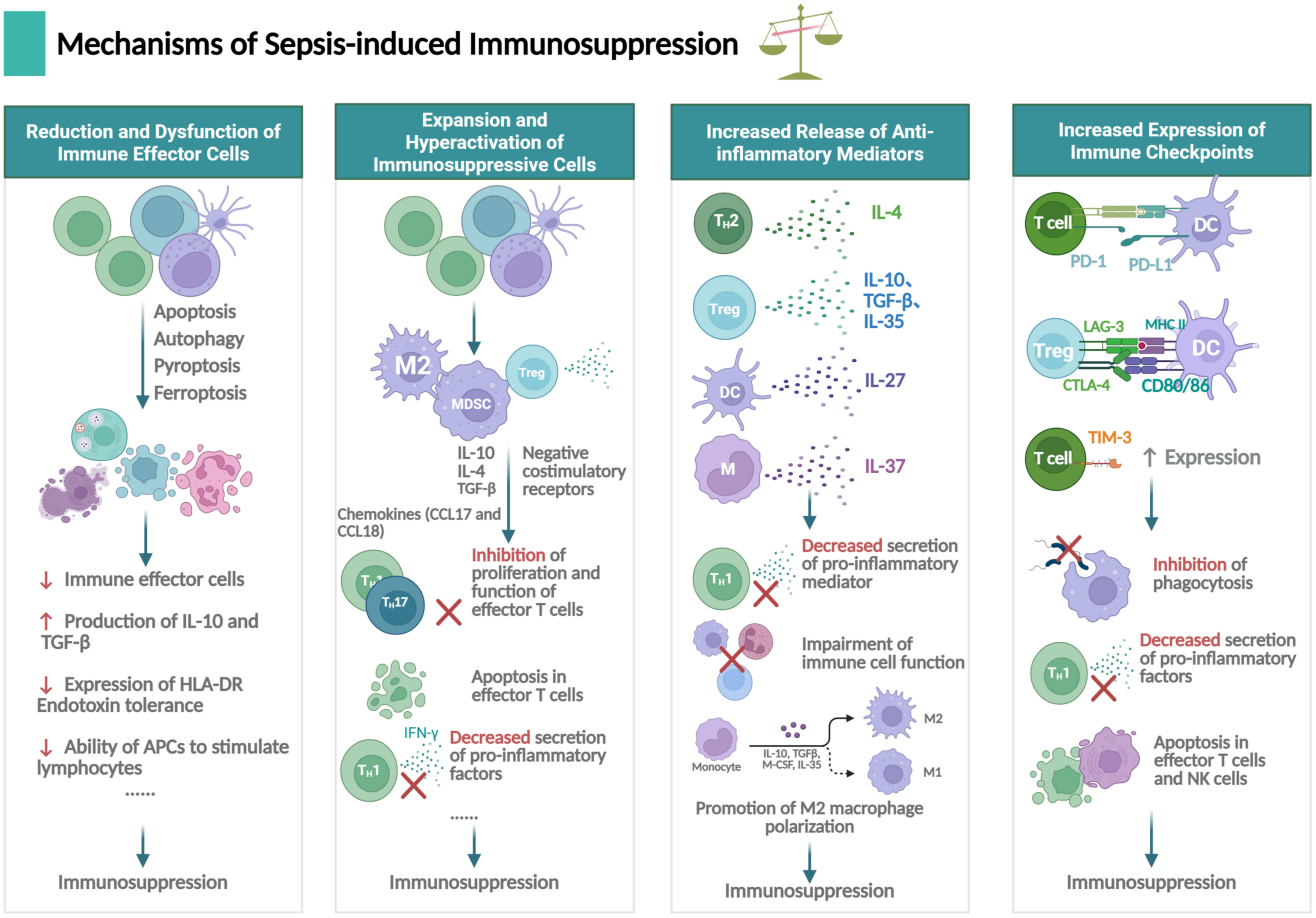
Mechanisms underlying immunosuppression in sepsis. Sepsis-induced immunosuppression involves dysfunction of immune effector cells, expansion of immune suppressive cells, secretion of anti-inflammatory mediators and expression of immune checkpoints, primarily concerning neutrophils, T lymphocytes, B lymphocytes, macrophages and dendritic cells (DCs), et al. The figure was created via BioRender (https://BioRender.com).

The phenomenon of “septic immunosuppression” was first identified by Volk et al. in 1996 (18), and studies on the mechanisms of “sepsis immunosuppression” emerged since then. Over the past decades, research into sepsis-induced immunosuppression has mainly focused on the dysregulation of immune effector cells and their failure to defend against infection, leading to increased susceptibility to secondary infections and death (5, 19). In recent years, with deeper understanding of the mechanisms, numerous studies have shown that sepsis-induced immunosuppression originates from the disorder of innate and adaptive immunity, characterized by the death and dysfunction of immune effector cells, overproduction of immunosuppressive cells, and release of anti-inflammatory cytokines. Increased expression of immune checkpoint molecules further aggravates immunosuppression (4, 20). Below, we comprehensively summarize the well-known existing studies on the mechanisms of sepsis-induced immunosuppression, with an overview shown in **Figure 3**.

### 2.1 Reduction and dysfunction of immune effector cells

In sepsis, the number of immune effector cells is often severely reduced, leading to immunosuppression and exacerbation of infections (21), which mainly involves apoptosis, autophagy, pyroptosis and ferroptosis.

Apoptosis is a regulated form of cell death that removes damaged cells and maintains homeostasis under physiological conditions (22), which is particularly prominent in CD4+ T cells, CD8+ T cells, B cells, natural killer (NK) cells, and follicular DCs. In the past decades, apoptosis in sepsis-related immunosuppression has gained much attention. Lymphopenia due to apoptotic loss is a common characteristic of septic patients (23). Postmortem analyses reveal profound splenic T cell depletion with concurrent PD-L1 upregulation in sepsis fatalities compared to non-infectious mortality controls (24), which may be attributed to the increased levels of cytochrome C, Bim, caspase-3, caspase-8, and caspase-9, while decreased expression of B-cell lymphoma/leukemia-2 (Bcl-2) (25). Likewise, studies have shown that macrophage apoptosis is significantly increased in sepsis, accompanied by the activation of signaling pathways involving Fas/FasL and TNF-related pathways (26). Consequently, these apoptotic cells are engulfed by macrophages and cleared from the inflamed area, triggering the production of IL-10 and TGF-β, decreased expression of HLA-DR, and endotoxin tolerance, all of which impair the ability of antigen-presenting cells (APCs) to stimulate lymphocytes (27, 28). Currently, the anti-apoptotic strategies have been successfully proven to reduce mortality after sepsis in mice (29), indicating that inhibiting apoptosis may be a strategy to restore immune function against infection.

Autophagy, a catabolic process involving lysosomal degradation and recycling of cytoplasmic components, can be either cytoprotective or induce cell death (30, 31). In sepsis, lipopolysaccharide (LPS) activates selective autophagy through TLR4-MyD88-dependent or MyD88-independent as well as NF-kB pathways (32). Autophagy plays a role in packaging pathogen components, while excessive autophagy can reduce inflammation, and prevent DAMP and PAMPs from binding to PRRs, thereby suppressing immune activation during sepsis (33). Additionally, autophagy can promote sepsis-induced apoptosis of T lymphocytes and reduce the inflammatory response by negatively regulating the abnormal activation of macrophages (34, 35). However, mice with a reduced autophagy capacity in lymphocytes due to cell-specific deletion of *Atg5* or *Atg7* showed an increased mortality together with higher release of the anti-inflammatory IL-10 and immune dysfunction in abdominal sepsis, suggesting that impaired autophagy can whereas contribute to immunosuppression (36). Therefore, the effect of autophagy on the immune status of sepsis may vary according to the stage.

In recent years, newly identified programmed cell death such as pyroptosis and ferroptosis, have also been found to be induced during sepsis and to play pivotal roles in sepsis-induced immunosuppression (37). Pyroptosis is a form of inflammatory cell death primarily mediated by caspase-dependent gasdermin-D (GSDMD) activation (38), characterized by loss of membrane integrity, cell swelling, rupture, and subsequent release of pro-inflammatory mediators (22). During sepsis, immunocyte pyroptosis may exert biological effects through classical pathways: Caspase-1 is activated, recognizes and cleaves GSDMD, after which the cleaved GSDMD forms pores in the cell membrane, ultimately triggering pyroptosis. Additionally, pyroptosis can occur through non-classical pathways involving caspase-4/5 in humans or caspase-11 in mice, which activate GSDMD to induce pyroptosis (39, 40). Pyroptosis may lead to the release of IL-1β, IL-18, and High Mobility Group Box-1 (HMGB1), recruiting immune cells to aggregate the inflammatory response, which are correlated with the hyperinflammation and subsequent immunosuppression (41). Notably, septic patients exhibit elevated caspase-1 expression in immune cells compared to healthy individuals (41). Furthermore, the costimulatory molecule GITR has been shown to promote immunosuppression in sepsis by enhancing NLRP3 inflammasome-mediated macrophage pyroptosis (42), whereas inhibition of the TMEM173-GSDMD-F3 pathway improves septic mice survival by blocking disseminated intravascular coagulation (DIC) (43), providing further evidence for pyroptosis in sepsis immunosuppression.

Similarly, ferroptosis is an iron-dependent cell death process driven by lipid peroxidation, involving iron overload, reactive oxygen species (ROS) generation, and accumulation of polyunsaturated fatty acids in phospholipids (44). During sepsis, infection induces upregulation of nuclear receptor coactivator 4 (NCOA4), which mediates selective ferritin autophagy, resulting in Fe³^+^ release and iron-dependent cell death (45). Emerging evidence indicates that ferroptotic cells release DAMPs, activating downstream signaling pathways that exacerbate sepsis-associated organ failure (46). Importantly, elevated serum iron levels, infection markers, and lipid peroxidation have been significantly correlated with increased long-term mortality and cognitive impairment in sepsis patients (47). Together, these evidences support the pivotal roles of ferroptosis in sepsis-induced immunosuppression.

### 2.2 Expansion and hyperactivation of immunosuppressive cells

#### 2.2.1 Regulatory T cells

The expansion of Tregs during sepsis suppresses the immune system by releasing anti-inflammatory cytokines, upregulating co-inhibitory receptors, and metabolic reprogramming from glycolysis to oxidative phosphorylation, which not only enhances their suppressive capacity but also correlates with long-term mortality in septic patients (48, 49). Mechanistically, Tregs inhibit effector T cell proliferation and function via direct secretion of inhibitory cytokines (e.g., IL-10 and TGF-β) and cell-contact-dependent suppression (50). Notably, Tregs can directly induce effector T cell apoptosis while impairing their antimicrobial activity, potentially mediated by Smad2/Smad3 signaling (51, 52). Furthermore, Treg-derived cytokines drive a feedforward immunosuppressive loop by promoting expansion of myeloid-derived suppressor cells (MDSCs) and establishing an immunosuppressive microenvironment, thereby exacerbating systemic immune paralysis in sepsis (53, 54).

#### 2.2.2 MDSCs

MDSCs constitute a heterogeneous cell population predominantly composed of immature myeloid cells with broad immunosuppressive activity targeting both innate and adaptive immunity (55). The pathological expansion of MDSCs in sepsis is driven by sustained production of inflammatory factors like IL-6 and TNF-α, and the activation of signaling pathways such as STAT3 and NF-κB (56, 57). The expansion of MDSCs is closely linked to chronic immune suppression in septic patients, affecting the severity and progression of sepsis and the incidence of secondary nosocomial infections due to their inhibitory effects on T cell proliferation and function (58, 59). Co-culture of MDSCs isolated from septic patients with T cells showed the impaired ability of antigen-driven T cells to proliferate and secrete proinflammatory factors such as IFN-γ (58). Notably, while immature myeloid cell numbers remain elevated for ≥ 6 weeks post-sepsis onset, only MDSCs acquired after the 2-week post-onset window exhibit potent inhibition of T lymphocyte proliferation and IL-2 synthesis, suggesting their pivotal role in maintaining chronic immunosuppression during the late phase of sepsis (60).

#### 2.2.3 M2 macrophages

Macrophages are functionally polarized into classically activated (M1) and alternatively activated (M2) subtypes, defined by their divergent roles in inflammation regulation. M1 macrophages exhibit pro-inflammatory phenotypes critical for pathogen clearance, whereas M2 macrophages display anti-inflammatory properties that facilitate tissue repair and resolution of inflammation (61). Notably, M2 macrophage polarization has emerged as a key driver of sepsis-induced immunosuppression. Mechanistically, during the late immunosuppressive phase of sepsis, M2 macrophages are activated by Th2 cytokines (IL-4 and IL-13), LPS, glucocorticoids, IL-10, IL-6, or TGF-β (62). In turn, these polarized M2 macrophages establish a self-reinforcing immunosuppressive cascade through massive secretion of IL-10 and TGF-β, thereby inducing host immune paralysis and predisposing to recurrent infections (63). Moreover, M2 macrophages express high levels of C-type lectins (CD206) and scavenger receptors (CD163), promoting the secretion of chemokines (CCL17 and CCL18) to recruit eosinophils, basophils, Th2, and Tregs, exhibiting an “anti-inflammatory cytokine profile” (64). Further research is needed to elucidate their complex regulatory network and functional mechanisms during sepsis.

### 2.3 Increased release of anti-inflammatory mediators

**Figure 4:**
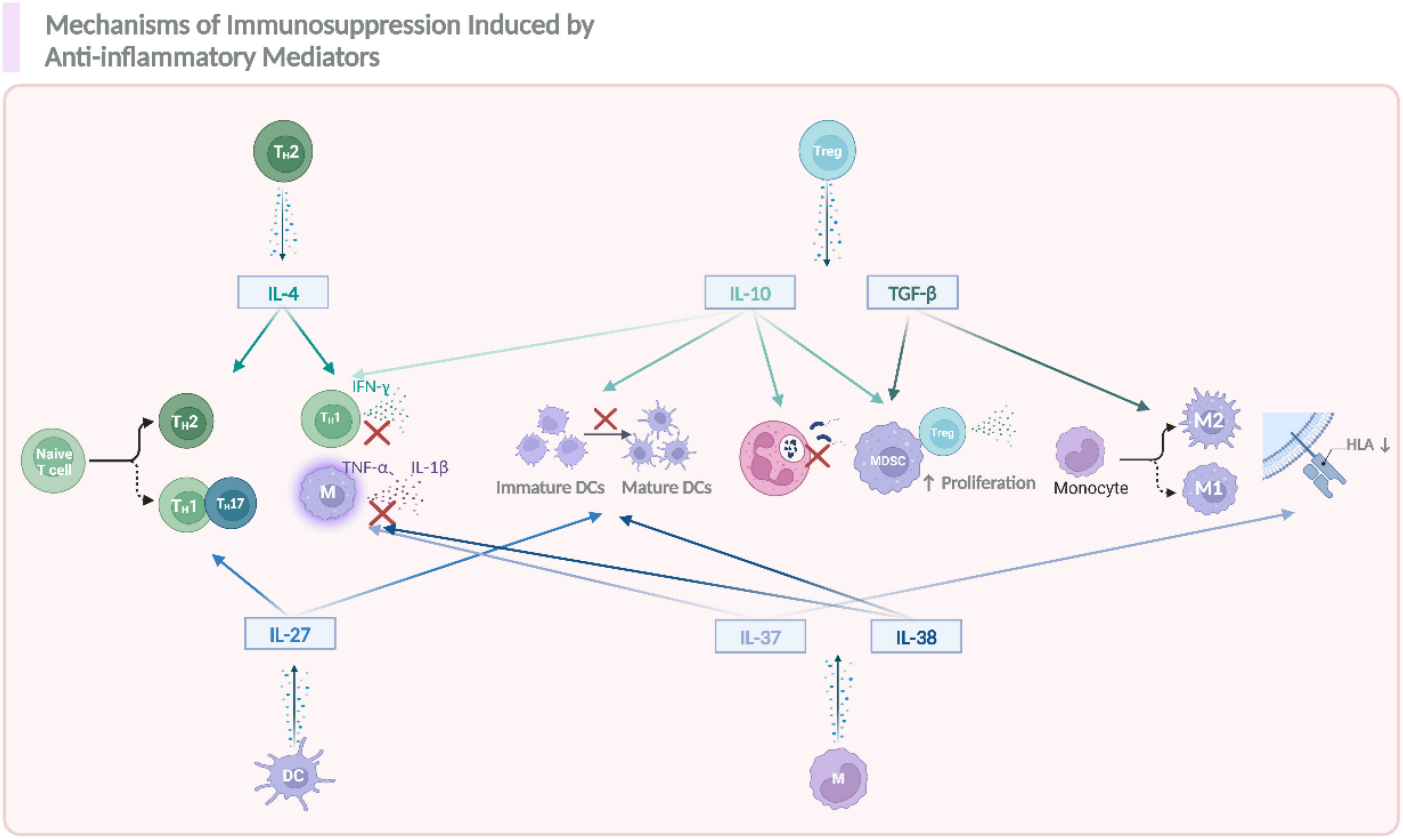
The role of anti-inflammatory cytokines in modulating immune responses and contributing to sepsis related immunosuppression. During the occurrence and progression of immunosuppression in sepsis, IL-4, IL-10, IL-27, TGF-β, IL-37, and IL-38 act as key anti-inflammatory mediators, promoting the differentiation of naïve T cells into T helper (Th) subsets, including Th2, and Th17. These mediators also suppress the activation of immune cells such as DCs, monocytes, and promote the polarization of macrophages toward an anti-inflammatory M2 phenotype. Additionally, they enhance the proliferation of Tregs and MDSCs, which further contribute to the suppression of inflammation. Notably, the reduction in HLA expression on immune cells also happened as a result of the activation of these anti-inflammatory signals. The figure was created via BioRender (https://BioRender.com).

Anti-inflammatory mediators play pivotal roles in the immunosuppression of sepsis, with specific actions including the inhibition of pro-inflammatory mediator secretion, the impairment of immune cell function, the promotion of M2 macrophage polarization, and the increase of immunosuppressive Tregs, *etc.* (65, 66). The most prominent cytokines in sepsis related immunosuppressive were IL-4, IL-10, IL-27, IL-37, IL-38 and TGF-β (28) (**Figure 4** **).**

IL-4 and IL-10 are crucial anti-inflammatory cytokines mainly secreted by Th2 cells, monocytes/macrophages and Tregs, et al, upon activation (67, 68). The release of IL-4 and IL-10 in sepsis suppress Th1 inflammatory response by reducing IFN-γ, TNF-α and IL-1β secretion by Th1 cells and inhibit Th1 cells polarization (65, 69). Specifically, IL-4 drives naive T cell differentiation toward Th2 lymphocytes, a process that can be blocked by anti-IL-4 antibodies (70); whereas IL-10 can exacerbate immunosuppression by promoting Treg and MDSCs production, inhibiting DCs maturation and neutrophil function, leading to impaired antigen presentation and pathogen clearance (71–73). Clinically, elevated plasma IL-10 levels demonstrate strong correlations with increased nosocomial infections and mortality rates, validating its central role in sepsis-associated immunosuppression (74). Collectively, these functions promote a Th2-biased immune response and weaken resistance to bacteria and viruses. TGF-β is a multifunctional cytokine belonging to the transforming growth factor superfamily. As for its anti-inflammatory properties, it plays a role in the induction of induced Treg cells (iTreg cells) from CD4+ T cells, alternative macrophage activation which maintain an anti-inflammatory phenotype, and act as an up-regulator of anti-inflammatory response (75, 76). IL-27, a dual-functional member of the IL-12 cytokine family, modulates sepsis pathogenesis through cell-type-specific actions on macrophages, DCs, and lymphocytes. While enhancing early inflammatory responses, IL-27 paradoxically suppresses Th1/Th17 differentiation and potentiates IL-10 production from Tregs and type 1 regulatory T (Tr1) cells during late-phase sepsis (77–79).

In recent years, the novel anti-inflammatory IL-35, IL-37, and IL-38 have also been implicated in sepsis immunosuppression, partly by inhibiting the release of pro-inflammatory mediators (80). Notably, Study have shown that IL-37 could significantly downregulate the expression of HLA-DR and CD86 in septic mice, inhibiting antigen presentation and indicating an immunosuppressive effect in sepsis (81). Clinically, septic patients exhibit elevated IL-37 levels that inversely correlate with inflammatory cytokine production and positively associate with immunosuppression severity (82). Previously, we have also shown that in COVID-19 patients developed into sepsis, IL-38 was increased and correlated with disease severity (83, 84), which consistent with mechanistic studies regarding IL-38 in promoting Treg expansion, while alleviating macrophage related pro-inflammatory responses in sepsis (85, 86).

### 2.4 Increased expression of immune checkpoints

Cell surface inhibitory immune checkpoints (also known as negative costimulatory molecules), including PD-1, PD-L1, CTLA-4, TIM-3, et al, are implicated in the immunosuppression of sepsis, by functionally inhibit phagocytosis and cytokine release of immune cells and promote T-cell exhaustion (87). As early as 2011, studies have reported increased expression of PD1-related molecules in sepsis patients, accompanied by decreased cytokine production, HLA-DR and CD28 expression, while increased activation of immunosuppressive Tregs (88, 89), providing direct evidence that PD1 is essential to the poor prognosis of sepsis. This concept is further supported by the finding that inhibition of PD-1 improved survival in septic patients (90). In terms of detailed mechanisms, upregulated PD-1 and PD-L1 in sepsis lead to suppression of T-cell function, and this interaction between PD-1 on T cells and PD-L1 on APCs (e.g., DCs) leads to T-cell exhaustion (87, 91). The PD-1 and PD-L1 interaction may also diminish myeloid cell function during sepsis, as indicated by decreased phagocytic capacity of both neutrophils and macrophages in sepsis patients (92). CTLA-4, an inhibitory receptor mainly expressed on activated T cells, inhibits T cell activity and play an immunosuppressive role in sepsis by binding to B7 molecules and CD80/CD86 (93). Recently, it was suggested that the upregulation of TIM-3, an immune checkpoint molecule in NKT cells, can promote NKT cell apoptosis, contributing to sepsis-related immunosuppression (68). TIM-3^+^CD4^+^ T cells show reduced proliferative ability and elevated expression of inhibitory markers compared to TIM-3^-^CD4^+^ T cells, and conditional deletion of TIM-3 in CD4^+^ T cells reduces mortality in response to sepsis (94). Additionally, TIGIT, an inhibitory receptor with Ig and ITIM domains, is predominantly expressed on T cells and NK cells; high expression of TIGIT has also been associated with immune dysfunction in sepsis (95). LAG-3 acts as an immunosuppressor in sepsis by inhibiting the activation and proliferation of T cells through binding to MHC II (96). However, so far, different studies have varying interpretations of the specific mechanisms of action of immune checkpoint molecules in sepsis, and the regulatory mechanism remains unclear, especially in patients with different types of sepsis.

### 2.5 Heterogeneity of immunosuppression in sepsis in terms of infection types

Sepsis is a dysregulated host response to infections caused by bacteria, fungi, viruses, or other pathogens, with distinct pathogenic mechanisms across pathogen types. In bacterial sepsis, endotoxins such as LPS bind to Toll-like receptor 4 (TLR4), thereby activating the NF-κB signaling pathway and driving the overproduction of anti-inflammatory mediators, a key contributor to immunosuppression (97). Bacterial pathogens may also evade immune clearance through biofilm formation, antioxidant enzyme synthesis, and exotoxin secretion, exacerbating immunosuppression (98).For fungal sepsis, beta-glucans on fungal cell walls activate the Dectin-1 receptor, triggering Syk and CARD9 signaling pathways that induce the production of both inflammatory and immunosuppressive cytokines (99). Concurrently, fungal infections impair antigen presentation by DCs and macrophages, while suppressing T cell and B cell activation (100). In viral sepsis, pathogens (e.g., SARS-CoV-2 or cytomegalovirus) activate RLRs and TLRs to initiate type I interferon (IFN-I) release (101). However, when evading the host immune system, virus also inhibits antigen presentation, cause a decrease in the activity of NK cells and cytotoxic T lymphocytes (CTLs), as well as blocking antibody production by B cells (102). Despite pathogen-specific mechanisms, bacterial, fungal, and viral sepsis share common immunosuppressive pathways, such as immune cell apoptosis induction and antigen presentation blockade. While our understanding of the pathogenesis of sepsis has significantly advanced in recent years, there is still much work to do to translate these new insights into effective treatments.

## 3 Impacts of immunosuppression on immune functions

**Figure 5:**
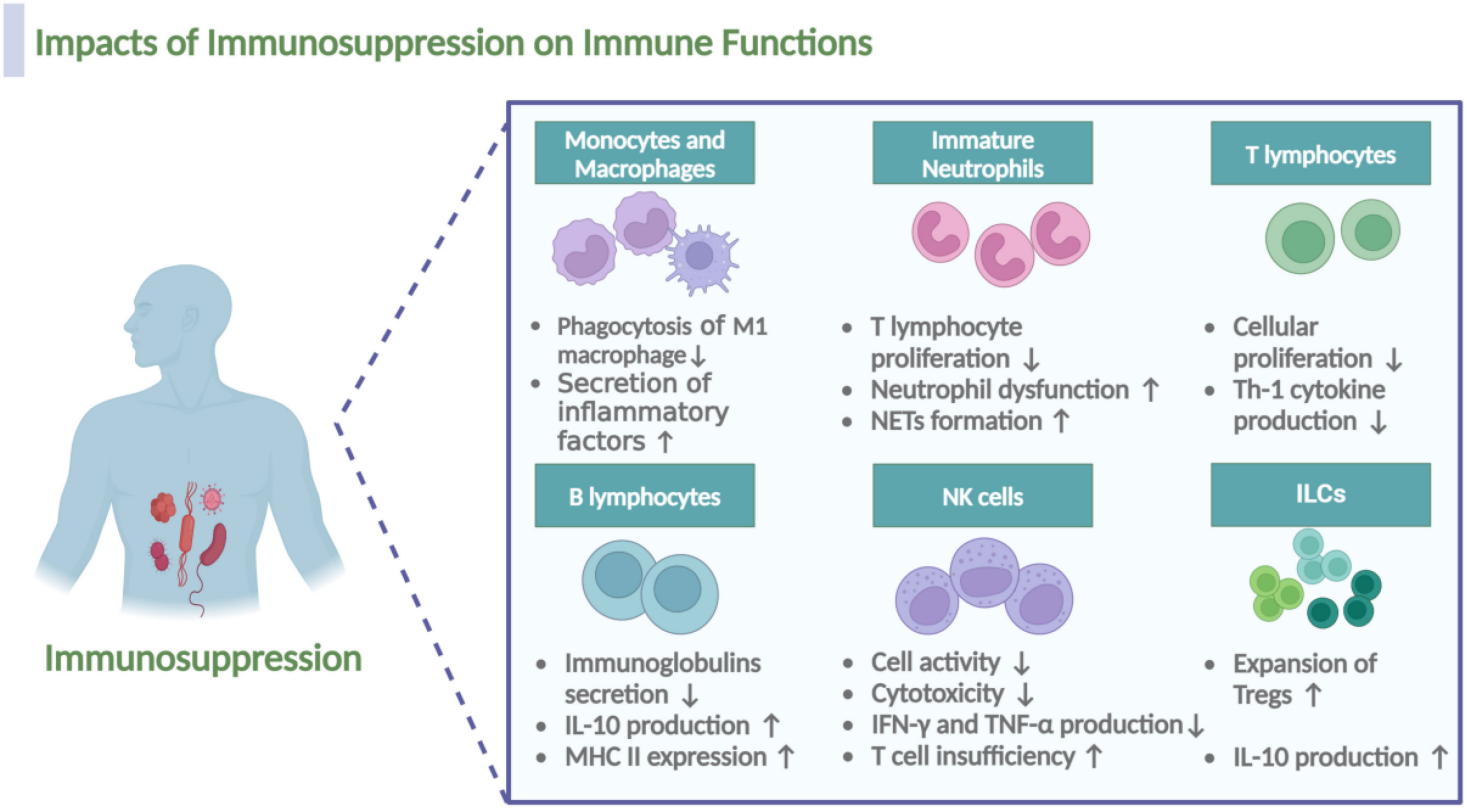
Impacts of immunosuppression on the immune cell functionality during sepsis. Immunosuppression during sepsis induces multifaceted dysfunction across immune cell populations. In macrophages, M1 subsets demonstrate impaired phagocytic capacity, while M2 macrophages exhibit upregulated secretion of anti-inflammatory mediators. Immature neutrophils acquire pathological functions, including suppression of T lymphocyte proliferation, exacerbation of neutrophil dysfunction, and potentiation of neutrophil extracellular traps (NETs) formation. T cells show reduced proliferative activity and diminished Th1 cytokine production, whereas B cells display decreased immunoglobulin secretion alongside elevated IL-10 production and MHC II expression. NK cells manifest compromised cytotoxicity coupled with reduced IFN-γ and TNF-α expression, collectively exacerbating T cell insufficiency. Notably, regulatory cell populations, including Tregs and the recently characterized ILCs, undergo expansion with concomitant increases in IL-10 production. The figure was created via BioRender (https://BioRender.com).

Sepsis directly or indirectly impairs the function of virtually all types of immune cells, involving macrophages, neutrophils, lymphocytes, NK cells, as well as intrinsic lymphoid cells (ILCs), the dysfunction of which may further aggravate immunosuppression of sepsis, forming positive feedbacks (**Figure 5**).

*Monocytes and Macrophages.* As a result of immunosuppression, the polarization of macrophage transitions from M1-type to M2-type, resulting in impaired antigen presentation and phagocytic dysfunction of M1 macrophages and increased secretion of anti-inflammatory factors of M2 macrophages, which partly depends on the decreased expression of MHC molecules, Tim4 and NALP-3 and the lack of costimulatory molecules (26, 103).

*Immature Neutrophils.* Notably, the expansion of immature neutrophils is also a characteristic and a result of sepsis related immunosuppression (104). In the first week following sepsis, patients exhibit profound changes in neutrophil characteristics; immature neutrophils, identified phenotypically by low expression of CD10 and CD16 (CD10^dim^CD16^dim^), are associated with increased early mortality after sepsis due to their immunosuppressive function (105, 106). Consistent with this observation, the existence of a subset of CD10^-^CD64^+^CD16^low/−^CD123^+^ immature neutrophils, that could be used for early identification of sepsis in patients was proposed (107). In murine models of sepsis, high expression of HMGB1 contributes to neutrophil dysfunction by limiting the activation of NADPH oxidase; neutrophils from septic patients, when cultured with plasma and anti-HMGB1, demonstrate a higher capacity to activate NADPH oxidase (108). Additionally, under the immunosuppressive stage, neutrophils also form NETs in sepsis, which, while trapping pathogens, also contribute to immunosuppression by interacting with Tregs (109).

*T lymphocytes.* In the early stages of sepsis, T lymphocytes typically exhibit an over-activated state, leading to the release of substantial amounts of inflammatory factors. As sepsis progresses in to the phase of immunosuppression, T cells gradually transition into an immunosuppressive state (48). Studies have revealed marked alterations in the mTOR pathway of T lymphocytes from septic patients, leading to a catabolic state characterized by a failure to induce glycolysis, oxidative phosphorylation, ATP production, or glucose uptake, ultimately impairing cellular proliferation (110). Notably, sepsis impairs the CD4+ T cell recall response and post-septic CD4+ T cells are highly glycolytic and exhibit a Th17 phenotype (111).

*B lymphocytes.* Regarding B lymphocytes, Manu Shankar-Hari et al. confirmed the hypothesis that B lymphocyte loss is a consequence of sepsis induced immunosuppression, involving different subsets, with the depletion and dysfunction of the memory B-cell population being the primary outcome of sepsis-induced immunosuppression (112). Notably, the impaired function of memory B cells and plasma cells results in an inability to secrete immunoglobulins in response to antigens (113). Additionally, surviving B lymphocytes exhibit an exhausted phenotype, characterized by increased IL-10 production and decreased MHC II expression (114).

*NK cells.* Impaired function of effector NK cells may also occur as a result of immunosuppression during sepsis. Secondary infections in patients who survived sepsis are often due to dysfunction in neutrophils, NK cells, and the inhibition of T-cells in the lung (115). As mentioned by Tang et al., NK cell activity is significantly impaired in patients with sepsis, potentially leading to a weakened immune response to infection and an increased risk of secondary infection (116). In septic patients, cytotoxicity of NK cells dramatically decreased, likely due to the reduction of CD3^-^CD56^+^NK cells; after stimulation with phorbol-12 myristate 13-acetate (PMA) and ionomycin, the production of IFN-γ and TNF-α by CD3-CD56+ NK cells in septic patients is also impaired (117).

*ILCs.* Notably, in recent years, the function of ILCs during sepsis induced immunosuppression has gained much attention. ILCs are a distinct class of lymphocyte subpopulations from T and B cells, including ILC1, ILC2, ILC3, and ILCreg. Helper ILC1s require T-bet for development and produce IFN-γ as their main effector cytokine; ILC2s depend on GATA-3 and produce “Th2” cytokines (IL-4, IL-5, IL-9, and IL-13); ILC3s depend on RORγt and secrete “Th17” cytokines (IL-17), or “Th22” cytokines (IL-22) (118, 119). It was found that ILC2 and ILC3 in the peripheral blood of sepsis patients was significantly reduced via apoptosis (120), and the reduction of ILCs may lead to immune escape of pathogens and deterioration of sepsis outcomes (119). A study showed that rhIL-7 immunotherapy could ameliorate sepsis-induced lung ILC loss, thereby ameliorating immunosuppression and survival in mice (121), providing further evidence for the role of ILCs in sepsis-related immunosuppression. Additionally, it was reported that IL-33 activates ILC2s, which produce IL-4 and IL-13 and drive M2 polarization of macrophages, resulting in the expansion of Tregs and immunosuppression via the production of IL-10 (122).

## 4 Immunosuppression-guided diagnostic biomarkers in sepsis

Over the past decades, numerous studies have identified biomarkers associated with sepsis-induced immunosuppression, significantly contributing to the diagnosis and treatment of sepsis. This paper aims to provide a comprehensive overview of both established and emerging immunosuppression-related biomarkers in sepsis, highlighting their clinical utility with actionable thresholds and assessing their clinical relevance. The relevant content is summarized in **Table 1**.

**Table 1:** Immunosuppression-related diagnostic biomarkers in sepsis.

Type of biomarkers	Name	Expression patterns in sepsis immuno-suppression	Thresholds	Reference
HLA-DR	mHLA-DR	Decrease	mHLA‐DR < 15,000 AB/C or percentage < 60%	(123)
Immune checkpoint markers	PD-1/PD-L1	Elevate	—	(92, 124, 125)
	CTLA-4	Elevate	CTLA-4 expression > 25% on CD4+ T cells	(126)
	TIM-3	Elevate	—	(94)
	IRAK-M	Elevate	> 2.32 ng/mL	(127)
	Galectin-1	Elevate	> 4.44 ng/mL	(127)
Immunocyte markers	Lymphocytes	Decrease	< 1.1 × 10^9^/L	(123)
	NLR	Elevate	> 4.18	(128)
	Treg	Elevate	Th17/Treg ratio < 0.69	(129)
	Siglec-F^+^ Neutrophils	Elevate	—	(14)
	HLA-DR^low^S100A9^high^Monocytes	Elevate	—	(130)
Cytokine markers	IL-10	Elevate	> 10 pg/mL	(131)
	IL-10LCR	Elevate	> 23.39 ng/mL	(132)
	IL-27	Elevate	> 5.0 ng/mL	(133)
	IL-37	Elevate	> 107.05 pg/mL	(134)
	TNF-α	Decrease	< 200 ng/L	(135, 136)

### 4.1 HLA-DR

HLA-DR expression serves as a robust indicator of monocyte antigen presentation capacity. In clinical studies, monocyte HLA-DR (mHLA-DR) has been employed as a marker for innate immunity. Notably, mHLA-DR levels in septic patients are significantly lower than those in healthy individuals (137). Reduced mHLA-DR levels are linked to immune dysfunction and poor prognosis in septic patients and critically ill COVID-19 Patients (138, 139). Clinically, mHLA-DR quantification using flow cytometry is recommended within 3–8 days after sepsis onset to guide immunomodulatory interventions (140). A significant correlation between SOFA score at admission and a decreased mHLA-DR expression at days 3–4 and 6–8 after onset was observed (141), with mHLA-DR < 15,000 AB/C or a percentage < 60% serving as a validated threshold for defining immunosuppression and predicting secondary infection risk (123). Furthermore, studies have demonstrated that mHLA-DR levels in 30-day non-survivors were significantly lower than in survivors, and septic patients with mHLA-DR ≥ 52.29% had a 6.798 times higher 30-day survival rate (142). This threshold may help stratify patients for targeted therapies. Importantly, septic patients with decreased mHLA-DR levels often do not return to normal until six months post-discharge, a pattern observed in patients with poor prognoses (143). Taken together, consensus now exists for considering low-monocyte mHLA-DR as a surrogate for sepsis-induced immunosuppression, making it the most extensively studied and validated biomarker in this domain.

### 4.2 Immune checkpoints

In sepsis, numerous studies have reported associations between increased PD-1 expression on T cells and PD-L1 expression on APCs with lymphopenia, T cell apoptosis, and mortality in sepsis patients. In 2016, a multivariate analysis found that increased monocyte PD-L1 expression was an independent predictor of mortality after sepsis, highlighting the significance of the PD-1 pathway in sepsis-induced immune dysregulation (124). Elevated PD-1 expression is also indicative of poor clinical outcomes in septic patients (125). The expression levels of PD-1 and PD-L1 in neutrophils and monocytes of septic shock patients are significantly higher than those in non-infected ICU patients and are positively correlated with sepsis severity and mortality (92). Therefore, dynamic monitoring of PD-1/PD-L1 expression may guide immunotherapy trials targeting these checkpoints in patients with sustained immunosuppression. Similarly, high CTLA-4 expression in sepsis is associated with immunosuppression, making it another biomarker for this condition (144). Actually, Cheng et al. demonstrated that CTLA-4 expression > 25% on CD4+ T cells was associated with a 3.2-fold increased risk of 28-day mortality, supporting its role in patient stratification for anti-CTLA-4 therapies for sepsis related immunosuppression (126). Through an RNA sequencing assay, Huang et al. found that the expression of TIM-3 on CD4+ T cells in septic patients with immunosuppression was significantly elevated and has been proposed as a prognostic marker for immune paralysis (94). Recently, Filippo Mearelli et al. examined 12 immune checkpoint markers in 113 patients with bacterial sepsis, and revealed that patients exhibiting elevated serum levels of IRAK-M and Galectin-1 (cutoff values: 2.32 ng/mL and 4.44 ng/mL, respectively) displayed clinical features of immunosuppression and poorer prognosis, with a 7-day mortality rate of 26% and an in-hospital mortality rate of 49% (127). Therefore, IRAK-M and Galectin-1, as inhibitory immune checkpoint biomarkers, could help identify a high-risk sepsis phenotype that might be suitable for enrollment in future checkpoint inhibitor trials.

### 4.3 Immunocytes

Immunocyte lymphopenia, characterized by reduced peripheral blood T and B lymphocyte counts, is a hallmark of acquired immune dysfunction in sepsis (145). Studies demonstrate that non-surviving sepsis patients exhibit a progressive decline in peripheral blood lymphocytes between days 2–7 post-onset, associated with a 3.5-fold increased mortality risk compared to survivors (146). Persistent lymphocyte counts below 1.0×10^9^/L serves as a clinical indicator of immune dysregulation, correlating with higher mortality rates and susceptibility to chronic infections (123). A retrospective observational study further revealed that a neutrophil-to-lymphocyte ratio (NLR) > 4.18 on day 7 predicts 28-day mortality with 81.94% specificity (128).

Additionally, patients with septic shock frequently develop immune collapse, characterized by reduced HLA-DR expression and elevated Tregs (119). Persistently high Treg levels are strongly linked to sepsis-induced immunosuppression and poor outcomes (147), with both Treg counts and the Th1/Th2 ratio proposed as biomarkers of immune suppression (123). Notably, aberrant Treg expansion often coincides with an inverted Th17/Treg ratio, indicating immunosuppression progression (148). This Th17/Treg imbalance is strongly correlated with disease severity with a ratio < 0.69 predicting mortality (AUC = 0.766) (129). Therefore, dynamic monitoring of lymphocyte counts can provide insights into the balance between innate and adaptive immunity, predict risks of secondary infections and mortality, and guide clinical interventions. Incorporating these thresholds into routine clinical laboratory panels could help identify high-risk patients requiring intensified surveillance.

Neutrophil dysfunction in sepsis includes reduced chemiluminescence intensity, while specific protein activations (e.g., CD88, TREM-1) may serve as diagnostic biomarkers for sepsis. Notably, diminished neutrophil CD88 expression predicts subsequent secondary infections and immunosuppression of sepsis (149). Recent study by Liao et al. identified Siglec-F^+^ neutrophils as immunosuppressive markers, with targeted depletion of this subset enhancing function of T lymphocytes and improving survival in preclinical models (14). The S100A8/A9 heterodimer, predominantly expressed in neutrophils and monocytes, emerges as a key immunosuppressive mediator in sepsis via impairing DCs maturation and promotes MDSCs accumulation (150). Transcriptomic analyses of monocytes from 29 sepsis patients and 15 healthy donors revealed upregulated S100A8 and S100A9 expressions linked to MDSC recruitment (59). Concurrently, a distinct monocyte subset (HLA-DR^low^S100A9^high^) was further identified as a driver of immunosuppression during sepsis; inhibition of this subset can markedly mitigate sepsis-induced immune depression, thereby providing a novel therapeutic strategy for the management of sepsis (130).

### 4.4 Cytokines

The immunosuppressive state in sepsis involves dysregulation of multiple cytokines, whose dynamic levels reflect disease severity and serve as diagnostic/prognostic biomarkers (151). During the immunosuppressive phase of sepsis, anti-inflammatory mediators typically demonstrate progressive elevation. Clinical evidence suggests that longitudinal IL-10 monitoring from day 1 to day 3 post-onset shows prognostic value for septic outcomes (131), while IL-10/lymphocyte ratio (IL-10LCR) at a cutoff of 23.39 ng/mL predicts 28-day mortality with 70.0% sensitivity and 74.4% specificity (132). In addition, using a cutoff of 5.0 ng/mL, the specificity of IL-27 to diagnose bacterial infection in immunocompromised septic patients reached 94% (133); and a cutoff value of IL-37 for predicting 28-day mortality in septic patients has been suggested as 107.05 pg/mL (134). Conversely, monocytes isolated from immunosuppressed sepsis patients exhibit markedly reduced production of pro-inflammatory cytokines, including TNF-α, IL-1β, IL-6, and IL-12 (135). Notably, Ploder et al. revealed persistent TNF-α suppression in circulating monocytes from non-surviving sepsis patients identifies it as a reliable immunosuppression marker (136). Mechanistically, LPS-stimulated monocytes from immunosuppressed sepsis patients show significantly attenuated TNF-α secretion than controls, making monocytic TNF-α production below 200 ng/L under LPS stimulation serving as a diagnostic threshold for immunosuppression in sepsis patients (135). Notably, single-cytokine assessment was inadequate to fully diagnosis sepsis immunosuppression due to the lack of specificity, serial monitoring using combined panels (IL-10, TGF-β, IL-37, et al) is therefore recommended for serial monitoring to track immune trajectory in the future.

## 5 Advances in targeted immunomodulation for sepsis-induced immunosuppression

**Figure 6:**
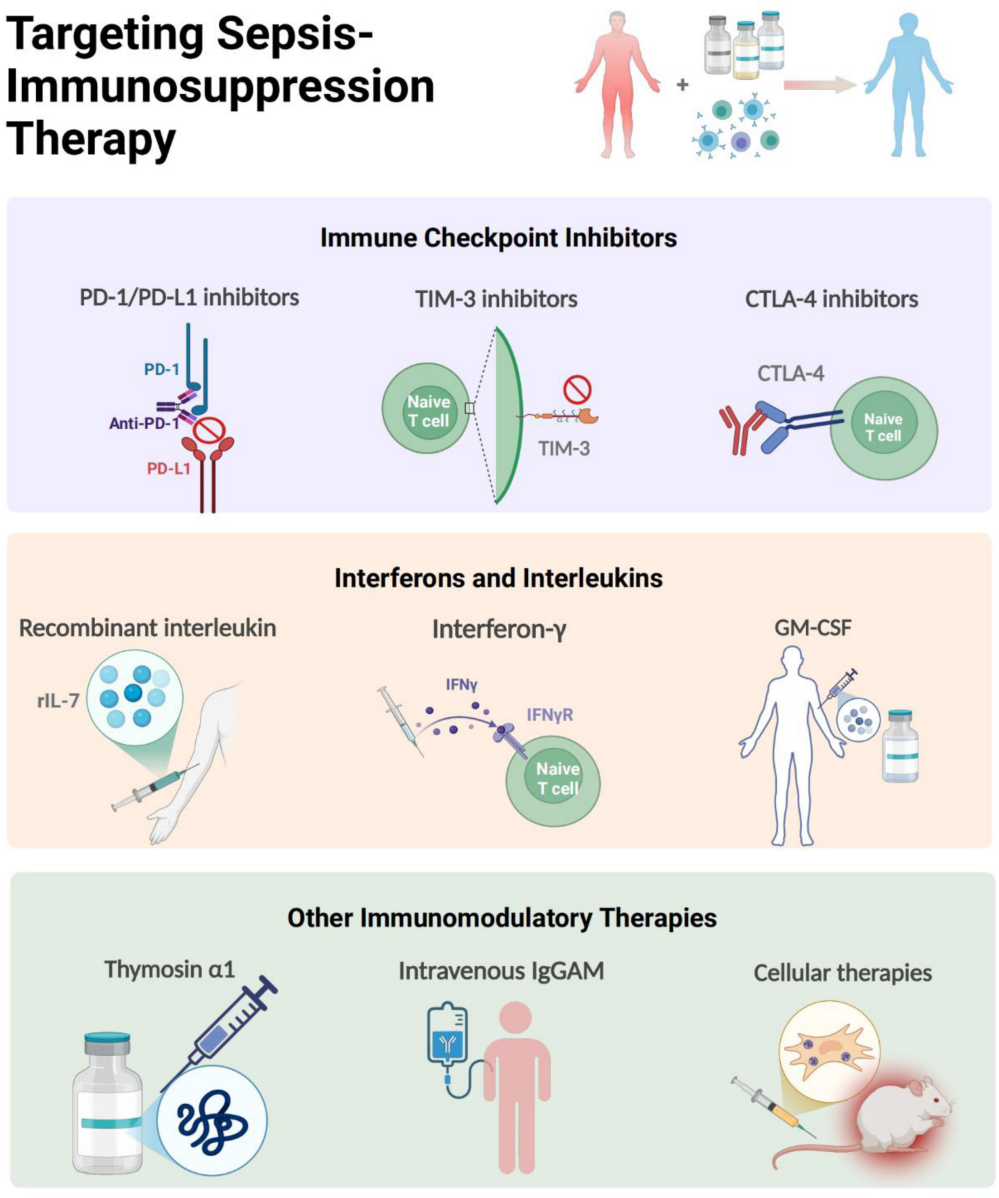
Targeting Immunomodulatory Therapy for Sepsis-immunosuppression. The immunomodulatory strategies of sepsis-induced immunosuppression mainly include immune checkpoint inhibitors, interferons and interleukins (IL-7, GM-CSF, IFN-γ, Thymosin alpha 1), MSCs and intravenous immunoglobulin. The figure was created via BioRender (https://BioRender.com).

Despite the failure of numerous clinical trials, some promise likely still exists in using anti-inflammatory treatment strategies in the early hours after the onset of sepsis such as IL-1 receptor blockade or anti-TNF treatments based on the identification of those that could survive from anti-inflammatory therapies in combination with antibiotics and resuscitation (152, 153). Nevertheless, most patients who survive the critical stage may eventually die of immunosuppression, and for those who developed into immunosuppressive conditions, immune-stimulation is therefore promising to revitalize the immune system to allow clearance of initial infectious foci or to fight secondary infections (123), and the field now increasingly focuses on reversing immunosuppression in later stages, a critical frontier where immune checkpoint inhibitors, cytokine therapies, as well as cell therapy like mesenchymal stem cells (MSCs), et al. show transformative potential. Here, we focus on the research of immunomodulatory therapy and provide a detailed summary of developments over the past decades, as depicted in **Figure 6**.

At the vanguard of this revolution are PD-1/PD-L1 axis inhibitors, which have progressed from murine sepsis models demonstrating restored T-cell functionality to pioneering human trials. In tested septic mice, 4-octyl itaconic acid modulates immune homeostasis in sepsis by activating Nrf2 and negatively regulating PD-L1, which may provide new therapeutic strategies (154). The landmark phase 1b randomized study by Hotchkiss et al. established the safety profile of the PD-1 inhibitor nivolumab and the PD-L1 inhibitor BMS-936559 in septic patients (90, 155), with *ex vivo* analyses revealing anti-PD-1 antibodies rescue exhausted CD8+ T cells by restoring IFN-γ production (156), a finding corroborated by parallel discoveries in TIM-3 and LAG-3 knockout mice models, showing that knockout of TIM-3 and LAG-3 reduces immunosuppression-related mortality of septic mice (94, 96). Notably, the use of TIGIT antibodies can restore the function of T cells from sepsis patients *ex vivo*, suggesting that blocking TIGIT may be a new approach for the immunotherapy of sepsis (157). However, these immunoadjuvant therapies are still under investigation, warranting large scale clinical trials, and whether they are promising in septic patients still has a long way to go.

Complementing checkpoint inhibitors are cytokine-based strategies undergoing clinical validation. Recombinant IL-7 emerges as a particularly promising candidate, demonstrating capacity to reverse sepsis-associated lymphopenia through mTOR pathway modulation (110), while showing excellent tolerability in randomized trials (158). In a prospective, randomized, double-blind, placebo-controlled trial involving 27 patients with septic shock and severe lymphopenia, rhIL-7 induced a three- to fourfold increase in absolute lymphocyte counts and circulating CD4^+^ and CD8^+^ T cells that persisted for weeks (159). Therefore, for those septic patients with severe lymphocyte alterations, rhIL-7 therapy is recommended to be one of the most promising potential adjuvant treatments. Moreover, in a recent completed multicenter and multinational, double-blind, double-dummy randomized clinical trial involving 280 patients with sepsis, the rhIL-7 was used to monitor the individualized immunomodulation therapy for those with immunoparalysis (160). The therapeutic effect of IFN-γ in septic patients has been recently validated. As early as in 2002, clinical studies has shown that inhaled IFN-γ treatment led to recovery of HLA-DR expression in alveolar macrophages and decreased incidence of ventilator-associated pneumonia (161). Later, a case report found that IFN-γ therapy combined with nivolumab (an anti-PD1 antibody) was effective in restoring immune function and eliminating invasive infection (162); and it was also being investigated in a phase 2 clinical trial to test whether rhIFN-γ would reverse the hypoinflammation and restoration of immune dysfunction of septic patients (163), and but no randomized, controlled trials have tested IFN-γ therapy in ICU patients to date. Similarly, GM-CSF (sargramostim) has transitioned from early clinical trial in 2002 and 2009 showing improved monocyte HLA-DR expression (164, 165), to recent multicenter trials in reporting reduced ICU acquired infections of immunosuppressed patients (166), proving further evidence that GM-CSF thus represents a promising immunoadjuvant therapy in patients with sepsis, although larger randomized controlled trials are now warranted to confirm these initial results. Notably, the ongoing SepTIC trial that includes investigation of sargramostim for improving outcomes in a high-risk subset of patients admitted to the ICU with sepsis starting in 2023 (167), would help exemplify the field’s growing sophistication in targeting immune paralysis across diverse populations.

The therapeutic arsenal continues expanding with novel modalities like thymosin α1 (Tα1) and IgGAM. A randomized controlled study showed that patients treated with Tα1 exhibited reduce mortality by 18% than the control group, accompanied with reduced mechanical ventilation time and ICU stay (168). In addition, IgGAM, a class of multi-component immunoglobulins rich in IgA and IgM, exerts therapeutic effects in sepsis primarily through mediating opsonization, neutralizing antigens, and regulating Fc receptor expression. The related drug Pentaglobin has demonstrated beneficial effects on sepsis in clinical trials (169, 170). However, the use of IgGAM is still controversial, with risks of various adverse effects and a lack of clear biomarkers to guide its accurate administration. Currently, a clinical trial based on serum IgM titers for IgGAM treatment in patients with septic shock is ongoing (NCT04182737) (171). Hopefully, as an adjuvant therapy, IgGAM may be a potentially effective immunotherapy for treating sepsis patients with immune-paralysis. Perhaps most intriguing therapies in the immunomodulation of sepsis are cellular therapies. MSCs not only mitigate organ damage via NLRP3 inflammasome suppression (172), but also synergize with antimicrobials through extracellular vesicle-mediated immunomodulation (173, 174). A Phase I open-label dose-escalation safety trial evaluating the efficacy of advanced MSC-based therapy in septic patients has enrolled 11 participants, though results remain unpublished (175), this multimodal approach epitomizes the field’s progression from single-target interventions to systems-level immune reprogramming.

## 6 Conclusions and perspectives

Over the past decade, researchers have made substantial progress in elucidating the mechanisms underlying sepsis-induced immunosuppression, including the identification of novel biomarkers for immune monitoring (e.g., dynamic changes in HLA-DR expression and lymphocyte function) and the development of immunomodulatory therapies aimed at improving clinical outcomes. Nonetheless, there are still limitations in the current studies. A primary concern is the continued reliance on animal models that inadequately replicate the biphasic immunological transition from initial hyperinflammation to subsequent immunosuppression observed in human beings. This fundamental discrepancy likely underlies the recurrent translational failures of immunotherapies that showed efficacy in rodent models but proved ineffective in human trials, such as early TNF-α inhibition strategies. Furthermore, while observational studies have established associations between immunosuppression and secondary infections in sepsis patients, the absence of controlled intervention trials leaves a pivotal question unresolved: Do immunostimulants directly mitigate infection risks, or are they merely markers of preserved immune competence? Compounding this uncertainty, current diagnostic approaches relying on static biomarker measurements may provide incomplete assessments of immune status. These limitations have direct clinical implications, as negative trial results may reflect suboptimal treatment timing rather than inherent therapeutic inefficacy.

To address these challenges, future investigations should prioritize three key domains. First, future research must focus on addressing the pathophysiological heterogeneity of sepsis through multi-omics approaches integrating genomic, metabolic, and immune signatures, as the high dimension of multi-omics profiling has been used to reveal the complexity of sepsis immunity and inflammation, enabling simultaneous analysis of multiple levels of RNA, proteins, lipids, and metabolites (176). Second, to enhance the clinical relevance of preclinical findings for sepsis, studies should focus on elucidating the temporal evolution of immunosuppression using longitudinal bio sampling and advanced functional assays. Third, in the past years, AI-enhanced learning is gradually used to determine individualized treatment strategies for septic patients. Although the system is under development for bedside use in a prospective randomized clinical trial in the ICU (177), the tool has help physicians make decisions. Therefore, implementing machine learning-driven predictive models that synthesize real-time biomarker data with electronic health records may enable precision stratification of septic patients for time-targeted immunotherapies.

In summary, this review synthesizes current understanding of immunopathological cascade of sepsis-induced immunosuppression, encompassing mechanistic insights, biomarker discovery, and emerging therapeutic interventions. We propose that understanding the heterogeneity of sepsis, dynamic monitoring of disease progression, and applying precise and individualized therapy are goals of future research to improve the survival of immunocompromised septic patients.
